# Spatiotemporal Patterns of Contact Across the Rat Vibrissal Array During Exploratory Behavior

**DOI:** 10.3389/fnbeh.2015.00356

**Published:** 2016-01-05

**Authors:** Jennifer A. Hobbs, R. Blythe Towal, Mitra J. Z. Hartmann

**Affiliations:** ^1^Department of Physics and Astronomy, Northwestern UniversityEvanston, IL, USA; ^2^Department of Biomedical Engineering, Northwestern UniversityEvanston, IL, USA; ^3^Department of Mechanical Engineering, Northwestern UniversityEvanston, IL, USA

**Keywords:** whisker, trigeminal, mechanics, trigeminal ganglion, tactile, haptics, exploratory procedure, active touch

## Abstract

The rat vibrissal system is an important model for the study of somatosensation, but the small size and rapid speed of the vibrissae have precluded measuring precise vibrissal-object contact sequences during behavior. We used a laser light sheet to quantify, with 1 ms resolution, the spatiotemporal structure of whisker-surface contact as five naïve rats freely explored a flat, vertical glass wall. Consistent with previous work, we show that the whisk cycle cannot be uniquely defined because different whiskers often move asynchronously, but that quasi-periodic (~8 Hz) variations in head velocity represent a distinct temporal feature on which to lock analysis. Around times of minimum head velocity, whiskers protract to make contact with the surface, and then sustain contact with the surface for extended durations (~25–60 ms) before detaching. This behavior results in discrete temporal windows in which large numbers of whiskers are in contact with the surface. These “sustained collective contact intervals” (SCCIs) were observed on 100% of whisks for all five rats. The overall spatiotemporal structure of the SCCIs can be qualitatively predicted based on information about head pose and the average whisk cycle. In contrast, precise sequences of whisker-surface contact depend on detailed head and whisker kinematics. Sequences of vibrissal contact were highly variable, equally likely to propagate in all directions across the array. Somewhat more structure was found when sequences of contacts were examined on a row-wise basis. In striking contrast to the high variability associated with contact sequences, a consistent feature of each SCCI was that the contact locations of the whiskers on the glass converged and moved more slowly on the sheet. Together, these findings lead us to propose that the rat uses a strategy of “windowed sampling” to extract an object's spatial features: specifically, the rat spatially integrates quasi-static mechanical signals across whiskers during the period of sustained contact, resembling an “enclosing” haptic procedure.

## Introduction

The rodent vibrissal system is one of the oldest and most well-established models for the study of tactile exploration and sensorimotor integration (Richardson, 1909; Vincent, 1912; Simons, 1978; Guić-Robles et al., 1989; Carvell and Simons, 1990; Ahissar and Arieli, 2001; Diamond et al., 2008; Bosman et al., 2011). During exploratory behaviors, rats tap and brush their vibrissae in a rhythmic “whisking” motion against objects of interest (Welker, 1964; Woolsey et al., 1975; Simons, 1985; Berg and Kleinfeld, 2003). Vibrissal movements are tightly coupled to sniffing (Welker, 1964; Deschênes et al., 2012; Moore et al., 2013), as well as to head and snout movements (Welker, 1964; Hartmann et al., 2003; Mitchinson et al., 2007; Grant et al., 2009, 2012).

Behavioral experiments have demonstrated that rats can orient to an object with a 90° corner or to a flat wall within a single whisk (Mitchinson et al., 2007; Grant et al., 2009), and it is clear from even the most cursory observation that rats can navigate through an environment without making multiple whisks at each location. These abilities suggest that rats can obtain important spatial information about an object or surface on the time scale of a single whisk (~125 ms).

What is the spatiotemporal structure of tactile input during a rat's initial encounter with a novel surface? To date this question has been unanswered, because the small size and rapid speed of the vibrissae preclude measuring precise vibrissal-object contact sequences during natural behavior.

Here we used a laser light sheet to quantify—at the millisecond time scale—the complete pattern of vibrissal-object contact during the rat's natural exploratory behavior while simultaneously monitoring head movements. We capitalized on the particularly intense whisking that occurs when a rat is presented with a novel object or placed in a novel environment (Welker, 1964; Berg and Kleinfeld, 2003; Sellien et al., 2005). This approach enables us to capture and study the interplay of head and vibrissal movements during natural exploratory behaviors.

## Materials and methods

All experimental work involving animals was approved in advance by Northwestern University's Animal Care and Use Committee.

### Behavioral methods and experimental setup

**Figure 1:**
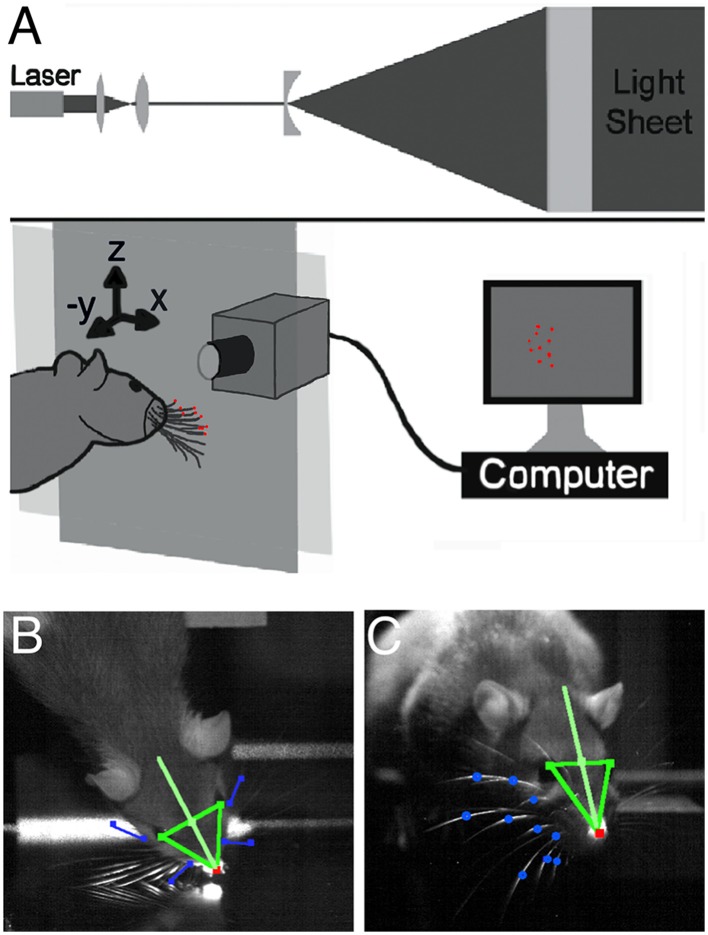
**Laser-light sheet setup and head and vibrissae tracking. (A)** An IR laser beam was passed through a set of optics to produce a collimated plane of light in front of a glass sheet (Towal and Hartmann, 2010). When the whiskers contacted the glass sheet, they interrupted the plane of the laser light, scattering points of light at the locations of contact with the glass. **(B)** One high speed video camera was used to track the nose, eyes, and rostral- and caudal-most whiskers in a top-down (“bird's-eye”) view. **(C)** A second high speed video camera tracked the nose, eyes, and all whisker-glass contact points in a head-on view (through the plane of the glass).

Subjects were five female Long Evans rats (2.5 months) naïve to the task. The behavioral setup is schematized in Figure 1A, and has been described in detail previously (Towal and Hartmann, 2010). Briefly, naïve rats—never before exposed to the setup—were placed on a platform from which they could stretch to explore a flat, vertical glass pane illuminated with a sheet of laser light. The glass pane was much larger than the rat's vibrissal array so that the rat could not touch the edges of the glass. Vibrissae could not touch the floor or ceiling. The only possible contacts were against the flat glass.

Rats were not rewarded for exploratory behavior; rather, the present experiments relied on their well-known natural tendency to intensely explore a novel object (Welker, 1964; Harvey et al., 2001; Berg and Kleinfeld, 2003; Sellien et al., 2005). Each rat was allowed to explore the glass for up to 10 s and then removed from the setup for at least 15 min. Three rats performed one trial, and two rats performed two trials.

Olfactory cues were controlled by carefully wiping the glass pane with alcohol between trials. Visual cues were controlled by operating in infrared illumination (940–980 nm), above the rat's visible range (Deegan and Jacobs, 1993). Audible signals were minimized in the room using an ultrasound detector (D230; Pettersson Elektronik AB, Uppsala, Sweden) to ensure that no high-frequency sounds were present.

### Tracking the head and whiskers

Two high-speed video cameras (1000 frames-per-second, Photron, San Diego, CA) recorded each trial. One camera captured a “bird's-eye” view (Figure 1B) and the second a “head-on” view through the glass pane (Figure 1C). Video recording was triggered by an IR sensor when the rat approached the glass and recording continued until the end of the trial. The temporal resolution of the analysis (1 ms) was set by the speed of the high-speed video cameras. The spatial resolution to which the positions of the vibrissae on the planar light sheet could be determined was 0.775 mm.

Standard image processing techniques permit two two-dimensional (2D) camera views to be merged into a single 3D image provided accurate measures are made of a 3D calibration tool of known shape and position (Hartley and Zisserman, 2003). Measurements of the necessary calibration points were made both before and after behavioral testing. To reconstruct 3D head position and orientation we semi-automatically tracked each rat's eyes and manually tracked the nose in both camera views, and the 2D trajectories were then merged into 3D.

In each frame of video, the rat's “head orientation vector” was defined as the vector from the mid-point between the eyes to the tip of the nose. A pitch of zero was defined as the angle at which the head orientation vector was perpendicular to the sheet and parallel to the ground. Head position (as determined by the 3D coordinates of the nose) and orientation were both filtered at 20 Hz. The 3D head velocity was calculated as the temporal derivative of the Euclidean distance between the location of the nose in two neighboring frames. We refer to instances “minimum head velocity” as those times at which the magnitude of this velocity vector is (close to) zero, that is, the head speed is minimized.

Angular positions of vibrissae were tracked in the top-down camera view and low-pass filtered at 25 Hz to remove tracking noise, consistent with standard techniques (Towal and Hartmann, 2006, 2008; Mitchinson et al., 2007).

### Quantifying vibrissal-object contacts

A five-step procedure was used to detect and quantify the locations of vibrissal-object contact (Towal and Hartmann, 2010). First, the two-dimensional (2D) locations of vibrissa-object contact on the plane of the glass sheet were automatically extracted from each video frame. Second, using techniques from particle-tracking velocimetry, we solved the correspondence problem for vibrissa-object contact points across sequential frames. Each contact point was given a unique identifier (e.g., vibrissa1, vibrissa2, etc.). The time-series of each whisker's contact point was low-pass filtered at 25 Hz. Third, a semi-automated program allowed users to manually identify each vibrissa based on its location in the array (e.g., A1, B2). After this initial identification, automated routines double-checked that vibrissae were in the correct order (e.g., A5 was closer to the nose than A4, and A5 was more dorsal than B5, etc.) and on the correct side of the face (e.g., left-side contacts were located to the left of the rat's nose). Vibrissa contacts that did not meet these criteria were flagged for manual correction. Finally, all trials were manually error-checked prior to analysis.

### Simulation of vibrissal contact and detach patterns

For the results shown in **Figure 6**, an anatomically accurate model of the rat head and vibrissal array was used to simulate vibrissal contact and detach patterns with a vertical wall (Knutsen et al., 2008; Towal et al., 2011). Whisking kinematics were governed by equations from Knutsen et al. (2008), as in previous work (Hobbs et al., 2015,in press). Briefly, the simulated rat was placed in the same position and orientation as the rat in the behavioral video. The protraction angle for the whiskers in a given column was set by linearly interpolating between the front whisker angle and back whisker angle from the behavioral data (Hobbs et al., in press). Right and left sides were treated independently. If any point along the simulated whisker penetrated the boundary of the vertical wall, the whisker was defined to have made contact.

## Results

### Overview

The present study analyzes the initial 1–3 s of head and whisker motion of five naïve rats as they explored a novel environment containing a flat, smooth, vertical glass surface. The results of this study therefore describe vibrissal-object contacts as the rat is “gaining an impression” of a novel object (between 2 and 21 whisks). The story that emerges from the rat's exploratory patterns is complex, spanning multiple temporal and spatial scales. To aid in understanding this complex story, this overview provides guideposts to the progression of the results that follow, divided into three main sections.

First, we characterize the rat's approach to the vertical surface and examine the effect of head movements on the vibrissal contact patterns. Consistent with previous studies that have emphasized variability in natural whisking profiles (Wineski, 1985; Carvell and Simons, 1990; Sellien et al., 2005; Towal and Hartmann, 2006; Mitchinson et al., 2007; Grant et al., 2009, 2012; Deutsch et al., 2012) we find that the whisk cycle cannot be uniquely defined because of asynchronies in right/left and rostral/caudal whisker motion, but that quasi-periodic (~8 Hz) variations in head velocity (head “dabs” Welker, 1964; Hartmann, 2001; Catania and Remple, 2004; Grant et al., 2012) represent a distinct temporal feature on which to lock subsequent analysis.

Next, we show that around times of minimum head velocity, the whiskers protract to make contact with the surface, and then sustain contact with the surface for extended durations before detaching. This behavior results in discrete temporal windows in which large numbers of whiskers are in sustained contact with the surface. We term these temporal windows the “sustained collective contact interval” (SCCI).

Finally, we ask three specific questions to quantify different spatiotemporal features of the SCCI. First, to what extent can the overall structure (on the timescale of around half a second) of the sustained contact patterns be predicted based only on information about head pose and the average whisk cycle? Second, what are the precise temporal sequences (on the timescale of milliseconds) of whisker-object contact and detach that demarcate the start and end of each SCCI? And third, what features characterize the motion of the whiskers on the surface during the sustained period of contact?

### Head position and orientation relative to the object is highly variable

**Figure 2:**
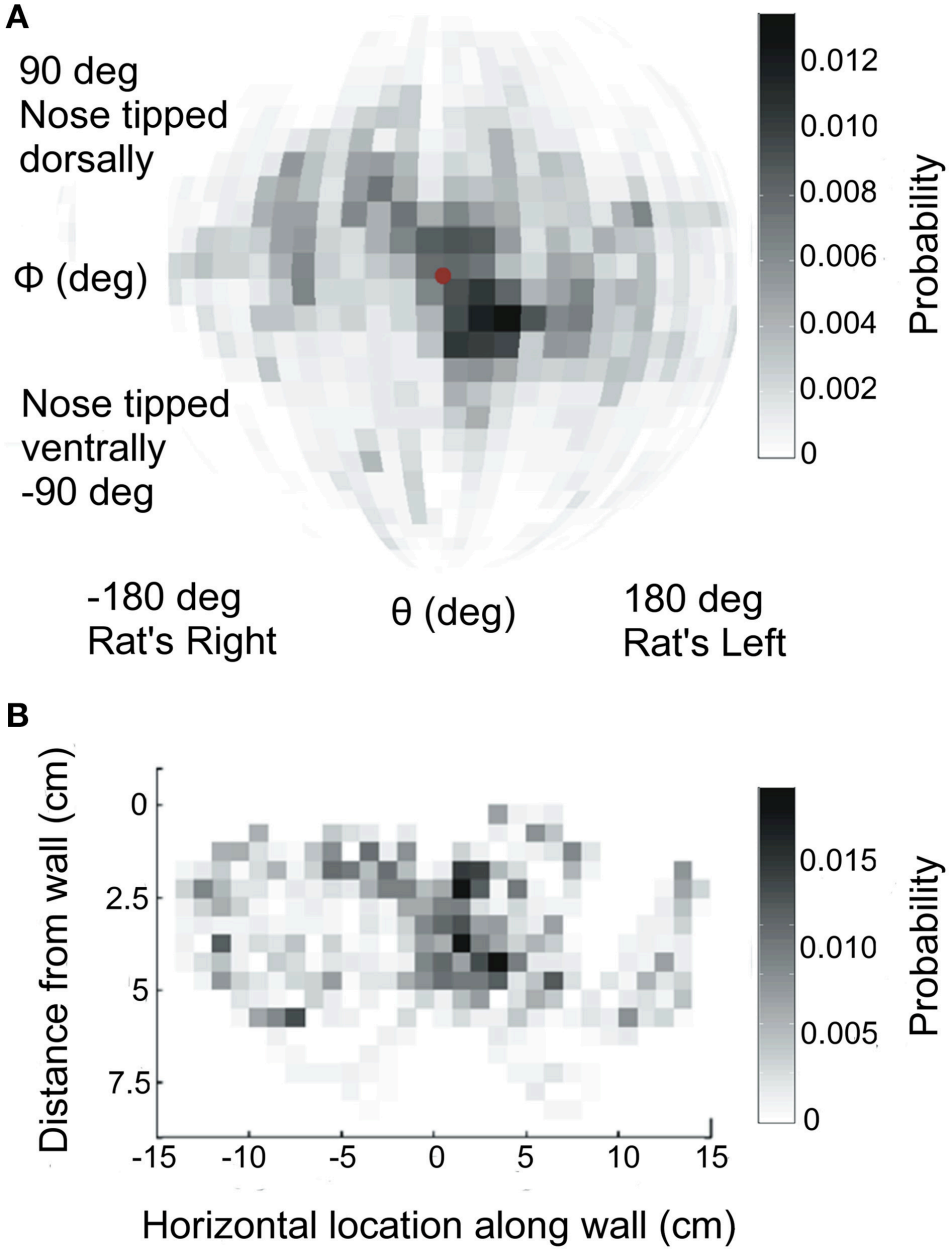
**Head orientation and position during exploration. (A)** Distribution of head orientations across all rats. Data have been mapped onto a sphere and are shown from the camera's point of view, looking through the glass pane at the rat. The center of the sphere (red dot) indicates a head orientation perpendicular to the glass pane. **(B)** Distribution of the nose locations relative to the glass pane, across all rats.

All rats stretched from the perch across the gap to explore the vertical glass sheet. Because the animals were completely free to move, they could approach the glass with their heads in different positions and orientations. Rather than controlling the approach poses, for example, by head restraint, the position and orientation of the head throughout the trial was carefully measured, as described in Materials and Methods. Rats approached the object with their heads in a variety of different poses (Figure 2A), and they maintained a variable distance from the object (Figure 2B).

**Figure 3:**
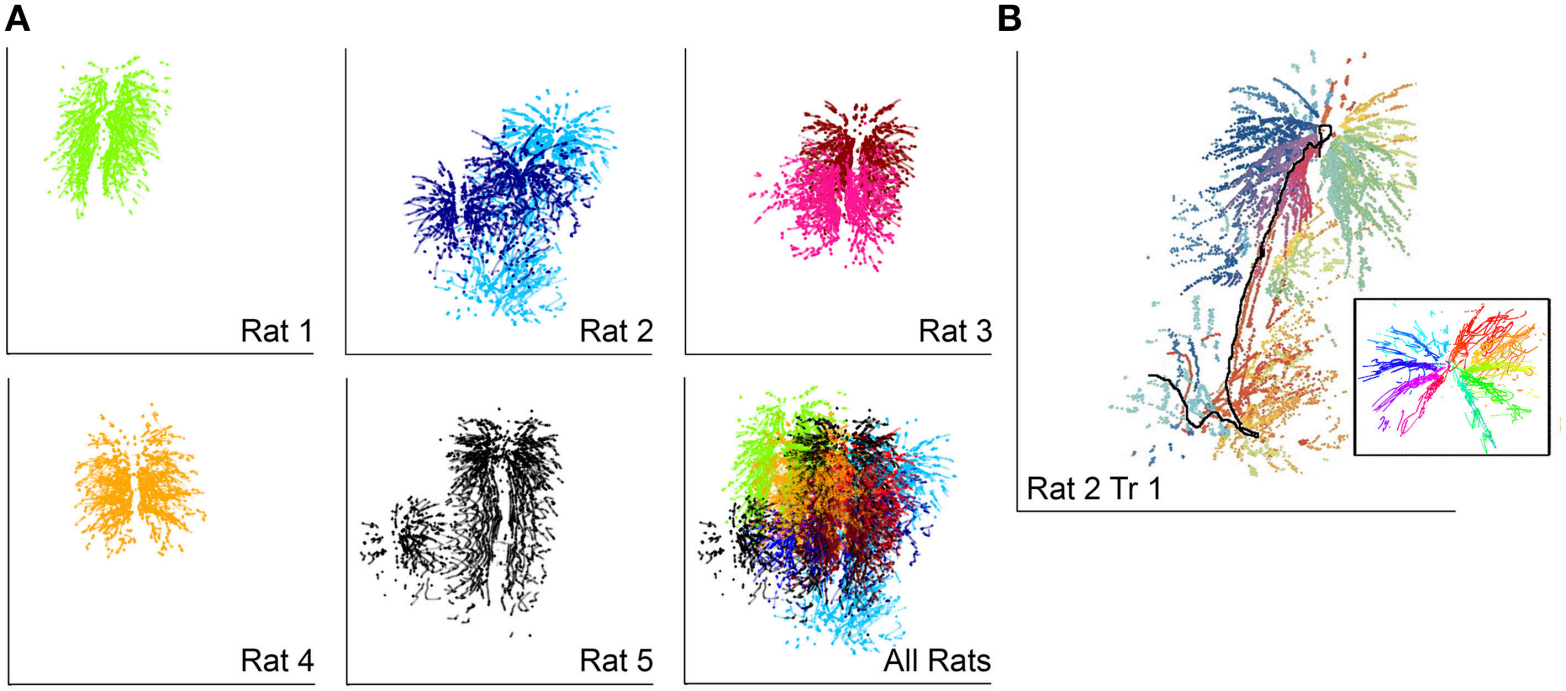
**Vibrissal-surface contact patterns depend strongly on head movements**. In all subplots horizontal and vertical lines indicate the full extent of the square glass wall (12 inches each side) **(A)** Each rat explored slightly different regions of the glass wall. Each dot represents the contact location of a single vibrissa. The different shades seen for Rats 2 and 3 correspond to Trial 1 and Trial 2. **(B)** Vibrissal-object contact points for the first trial of Rat 2. Vibrissa identity is indicated by color; rows are assigned similar colors. The black line indicates the trajectory of the rat's nose, which moved from top to bottom and was not in contact with the wall. The inset shows the contact pattern with the effects of head movement removed and the nose represented as a black dot at the center of the radiating pattern.

Patterns of vibrissal contact against the glass pane made by each of the five rats are shown in Figure 3A. Each rat explored only a fraction of the glass stimulus during any one trial, but collectively the rats explored almost the entire extent of the glass. In most (5/7) trials the rat began exploration by pitching its head increasingly upward, seen as a clear vertical trend in the pattern of contact. This behavioral strategy has previously been demonstrated to specifically increase the number of ventral vibrissa in contact with the wall, providing key information about the pitch of the surface relative to the rat's head (Grant et al., 2009; Hobbs et al., in press).

Notably, the complex patterns of vibrissal-surface contact illustrated in the five examples of Figure 3A result from the combined effects of both head and whisker movements. An example of the relative contributions of head and whisker movements is shown in Figure 3B, which re-plots the contact patterns for Rat 2 with each vibrissa uniquely colored. The inset to the figure illustrates the patterns of contact produced after subtracting the effect of head motion, revealing a clear “pinwheel” radial contact pattern. Each whisker falls into a reasonably fixed position relative to the head, so that the vibrissal rows form the branches of the pinwheel. It is clear, then, that in the absence of head movement, vibrissae fall into “slots”; coupling head movements to the whisk-cycle produces a far more complex pattern that also covers a far larger portion of the surface of interest. Vibrissa-object contact patterns from the exploratory bout shown in Figure 3B can be seen in Movie 1.

### The number of whiskers in contact with the wall covaries with head velocity and with the whisk cycle, but these three variables are not tightly locked

Given that the patterns of vibrissal-object contact depend so strongly on head movements, we began our analysis by quantifying head velocity. All five rats exhibited large fluctuations in 3D head velocity during tactile exploration.

**Figure 4:**
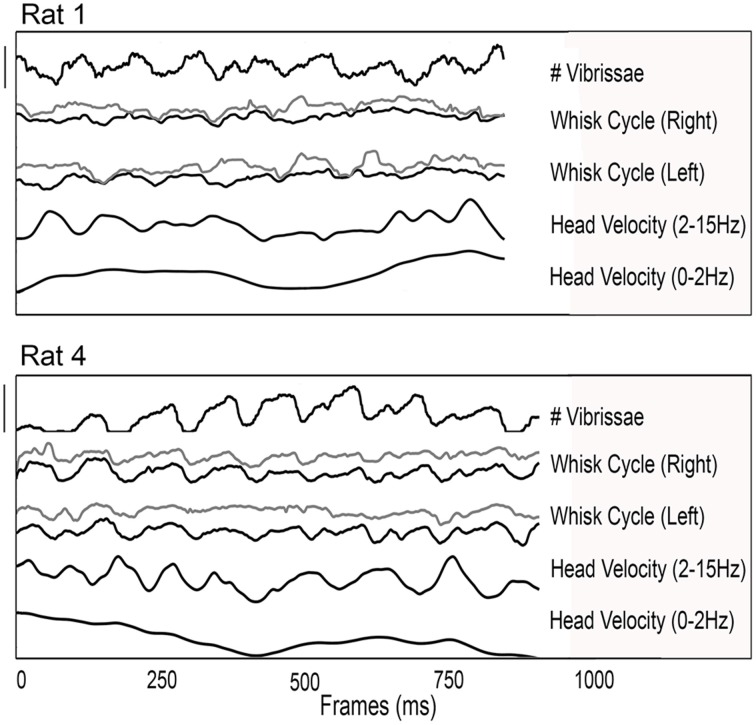
**Relationships between 3D head velocity, whisk cycle, and the number of vibrissae in contact with the object surface**. Examples from two rats are shown. In both plots, from top to bottom the traces show: the number of vibrissae in contact; the whisk cycle for whiskers on the right side (gray, rostral; black, caudal); the whisk cycle for whiskers on the left side (gray, rostral; black, caudal); the 3D head velocity filtered between 2 and 15 Hz; and the 3D head velocity filtered between 0 and 2 Hz. For both rats, it is clear that differences between rostral and caudal whiskers and right and left sides prohibit unique determination of the “whisk cycle” as the whiskers interact in complex ways with the surface. In contrast head motion tends to retain a quasi-periodic structure. Scale bar for Rat 1: Number of vibrissae: 25 vibrissae (range = 11–36); Whisk cycle (angular position): 140° (range = 40–180°); Head velocity (high pass filtered): 0.125 mm/ms; Head velocity (low pass filtered): 0.115 mm/ms. Scale bar for Rat 4: Number of vibrissae: 55 vibrissae (range = 0–55); Whisk cycle (angular position): 100° (range = 60–160°); Head velocity (high pass filtered): 0.125 mm/ms; Head velocity (low pass filtered): 0.115 mm/ms.

Previous studies have shown that head velocity is correlated with the whisk cycle under a variety of exploratory conditions (Welker, 1964; Hartmann, 2001; Catania and Remple, 2004; Grant et al., 2009), so we searched for this effect in our dataset. Two typical examples of exploratory behavior from two different rats are shown in Figure 4. In both examples, the 3D head velocity has been filtered into low (0–2 Hz) and high (2–15 Hz) frequency components. The low frequency component of the 3D head velocity is shown as the bottom trace of each plot and contains approximately equal contributions of motion both toward/away and parallel to the glass sheet. The higher frequency component of the 3D velocity is shown in the second trace from the bottom of each plot. At this time scale, the head velocity varies approximately with the protraction angle of the whiskers, shown for both right and left arrays and for rostral and caudal whiskers in the third and fourth traces of each plot. This result is consistent with those of previous studies showing that “dabs” of the snout co-vary with the whisk cycle (Welker, 1964; Hartmann, 2001; Grant et al., 2012).

The top-most traces in the two examples of Figure 4 represent the number of whiskers in contact with the glass surface. The number of whiskers in contact with the object covaries strongly with the whisk cycle, as well as with the 2–15 Hz component of the 3D head velocity.

The most salient feature of the two examples of Figure 4 is the strong ~8 Hz quasi-periodicity in three signals: head velocity, the whisk cycle, and the number of whiskers in contact. This ~8 Hz periodicity was found in these three signals for all trials of all rats. Although it is tempting to perform a correlation analysis between these signals, it is important to remember that two quasi-periodic signals in the same frequency range can appear correlated when they are actually not. In the present case, the correlation between the whisk cycle and the number of whiskers in contact with the surface yielded an *r*^2^-value of 0.59 ± 0.14, and in general the times of minimum head velocity tended to correspond to times of peak protraction. The correlation between head velocity and the number of whiskers was *r*^2^ = 0.55 ± 0.11. Visual inspection of the data, however, shows that the peaks and troughs of the 8 Hz signals shifted back and forth relative to each other; in other words, the signals are not strongly temporally locked.

These plots also highlight some of the difficulties in uniquely identifying a “whisk,” as already indicated in earlier publications (Wineski, 1985; Sellien et al., 2005; Towal and Hartmann, 2006, 2008; Mitchinson et al., 2007; Grant et al., 2009, 2012; Huet and Hartmann, 2014). The precise time of maximal whisker protraction and retraction is often ambiguous because of asynchronies between right and left sides and between caudal and rostral vibrissae. Additionally, features such as “double pumps” (Wineski, 1985; Towal and Hartmann, 2008; Deutsch et al., 2012) introduce secondary local maxima, often of magnitude comparable to the primary whisk, and often very different for caudal vs. rostral vibrissae. Finally, as vibrissae contact the object, their motion becomes increasingly complex (c.f., Deutsch et al., 2012), particularly if the vibrissae are dragged across the glass and remain in contact during multiple whisks.

In contrast, identification of peaks and troughs in the head velocity is more straightforward. Using times of minimum head velocity allows a single, unambiguous temporal marker to be identified that approximately covaries with the number of whiskers in contact. Subsequent analyses will therefore explore the timing of contacts and detaches relative to these times of minimum total head velocity.

### The times of vibrissal contact and detach relative to head velocity demarcate periods of sustained, collective contact

So far, results have shown that vibrissae are more likely to be in contact with the wall around the times when the head velocity is low, which also tends to correlate with peak protraction in the whisk cycle. These three variables—number of whiskers in contact, head velocity, and the whisk cycle—tend to covary but are not strictly temporally correlated. To further quantify this tendency, without making any assumptions about temporal coherence, we performed an analysis based on the following three parameters:

- “Number of contacts”—a vibrissa that was not previously in contact comes into contact with the wall
- “Number of detaches”—a vibrissa in contact in the previous frame ceases to be in contact
- “Number of vibrissae in contact”—the number of vibrissae touching the wall in a given frame

**Figure 5:**
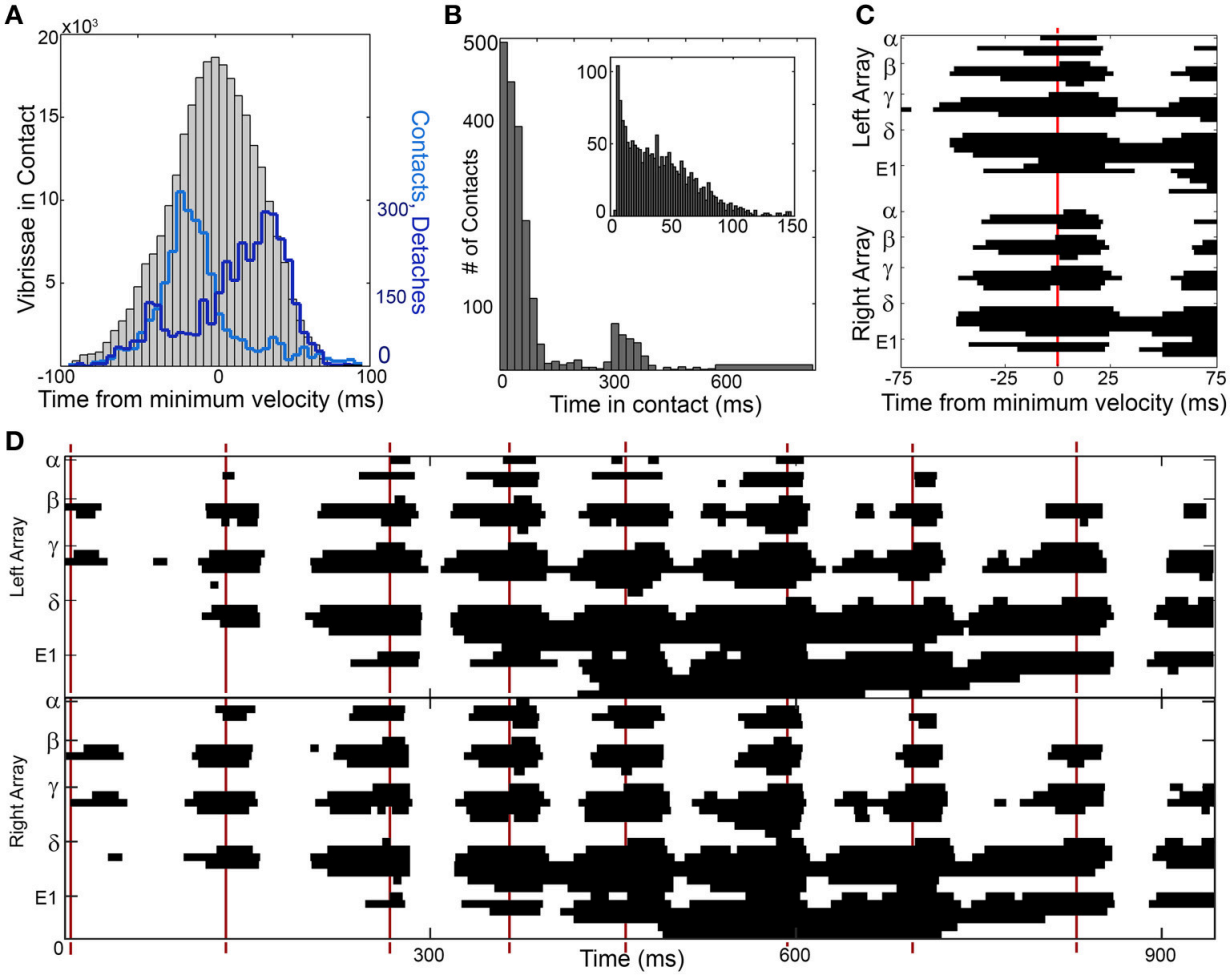
**Contact distributions around times of minimum head velocity. (A)** The number of vibrissae in contact with the wall (gray histogram) is normally distributed around the time of minimum head velocity (*t* = 0). The number of contacts (light blue line) peaks before minimum head velocity and the number of detaches (dark blue line) peaks after the minimum head velocity. **(B)** Vibrissae maintain contact with the glass surface for extended durations of time. The bimodal distribution is observed because whiskers often sustain contact for more than one whisk. The inset expands the portion of the distribution corresponding to contacts that last for less than one whisk (~150 ms). **(C)** One example of a sustained collective contact interval (SCCI) is seen in the vibrissal-object contact pattern generated by a single whisk, centered about a time of minimum head velocity. In this figure the y-axis is discretized into 62 rows. Each row of the figure illustrates the contact pattern of one whisker from either the right or left array. The whiskers are ordered by row and sub-ordered by column, so from top to bottom they are: α, A1 …A4, β, B1 …B5, γ, C1 …C6, δ, D1 …D6, E1 …E6. The x-axis is discretized into pixels with 1 ms time resolution. Each pixel in the figure is colored black if the whisker was in contact with the glass sheet at that time, and colored white if the whisker was not in contact with the glass sheet at that time. Most contacts precede minimum head velocity, detaches follow minimum head velocity, and the total number of vibrissae in contact is centered on minimum head velocity. **(D)** Contact pattern for an entire trial of contact. Patterns of sustained contact are centered around times of minimum head velocity, indicated by red vertical lines. Figure conventions follow those in **(C)**. The edges of the sustained contact are particularly well defined during the first half of the trial. They become less well defined in the second half of the trial, as the rat pitches its head downward, dragging the ventral whiskers along the glass. The pattern centered near 400 ms is the portion of the trial corresponding to **(C)**.

Figure 5A shows these counts distributed about the times of minimum head velocity across all rats. The number of vibrissae in contact with the wall is strongly peaked about times of minimum head velocity, following a Gaussian distribution with a standard deviation of 26.9 ms. Very similar distributions were also seen for each rat individually. The number of contacts peaks slightly before minimum head velocity while the number of detaches peaks after minimum head velocity. Thus, the whiskers are seen to come into contact as the head slows, generally sustain contact, and then detach as the head velocity increases.

Figure 5A also illustrates that the distribution of times when the vibrissae are in contact with the wall is broad, extending the full duration of the whisk cycle. The breadth of the distribution occurs in part because some whiskers tend to maintain contact with the wall for an extended period of time; Figure 5B further quantifies this duration of contact. The average contact lasts 96.6 ms, but some vibrissae remain in contact for upwards of 800 ms. For those contacts lasting 300 ms or less, the mean contact time is 53.5 ms, and for contacts lasting 150 ms or less, the mean contact time is 41.0 ms.

We can further explore the nature of these contacts, detaches, and sustained contacts by focusing on a single instance of minimum head velocity. The contact pattern around a time of minimum head velocity for Rat 4 is shown in Figure 5C. As suggested by the distributions of Figure 5B, the vibrissae make contact and then tend to sustain contact with the wall for extended durations.

We termed this period of sustained contact around the time of minimum head velocity the “SCCI,” to emphasize both its temporal duration (sustained) as well as the large number of vibrissae that make contact with the object (collective). The SCCI will be quantified in detail in the next three sections. We deliberately do not define a precise duration for the SCCI, because large numbers of whiskers can sustain contact for ±15 to ±35 ms depending on the whisk. The exact choice of window duration does not have any effect on any subsequent results.

Typical results when contact patterns are plotted across an entire trial are shown in Figure 5D. Although the exact timing of contacts and detaches varies from whisk to whisk, there is a clear SCCI surrounding each instance of minimum head velocity. The whiskers are seen to “scatter on” to the wall, remain in contact for an extended period of time, and then “scatter off” as they detach. This produced distinct temporal clusters of contact in the form of SCCIs for all whisks, on all trials, for all rats.

The results shown in Figure 5D leave us with three large open questions which are explored in the next three sections. First, what features of rat exploratory behavior explain the overall patterns of contact (on the timescale of seconds) seen as long sequences of SCCIs? Second, what is the detailed temporal structure of the contacts and detaches that demarcate the start and end of each SCCI as the whiskers “scatter on” and “scatter off” the vertical surface? And third, what is happening *during* the SCCI, when the whiskers sustain contact with the surface?

### The overall structure of contact patterns can be predicted from head pose and protraction angle

We first investigated the variables that determined the overall spatiotemporal structure of the contact sequence, on the timescale of multiple whisks. Recent work has shown that the identity of which whiskers have the potential to come into contact with a surface is largely determined by head pose alone (Hobbs et al., 2015,in press). Therefore, in the present dataset, we examined the extent to which head pose could predict the overall spatial structure of the contact patterns. To do this, a 3D model of the rat head (Towal et al., 2011; Huet and Hartmann, 2014; Hobbs et al., 2015,in press) was placed in the same position and orientation as the rat's head measured behaviorally, and then, at each head pose at each point in time, the whiskers were simulated to protract until as many vibrissae as possible made contact with the wall.

We emphasize that this first simulation, which models full protraction at each head pose, is intended to predict an upper bound on the spatial structure of contact. The simulation is not expected to predict any temporal features of contact. Nor is it expected to predict the exact whiskers that make contact, given that the rat does not have its whiskers fully protracted at all head poses at every point in time.

**Figure 6:**
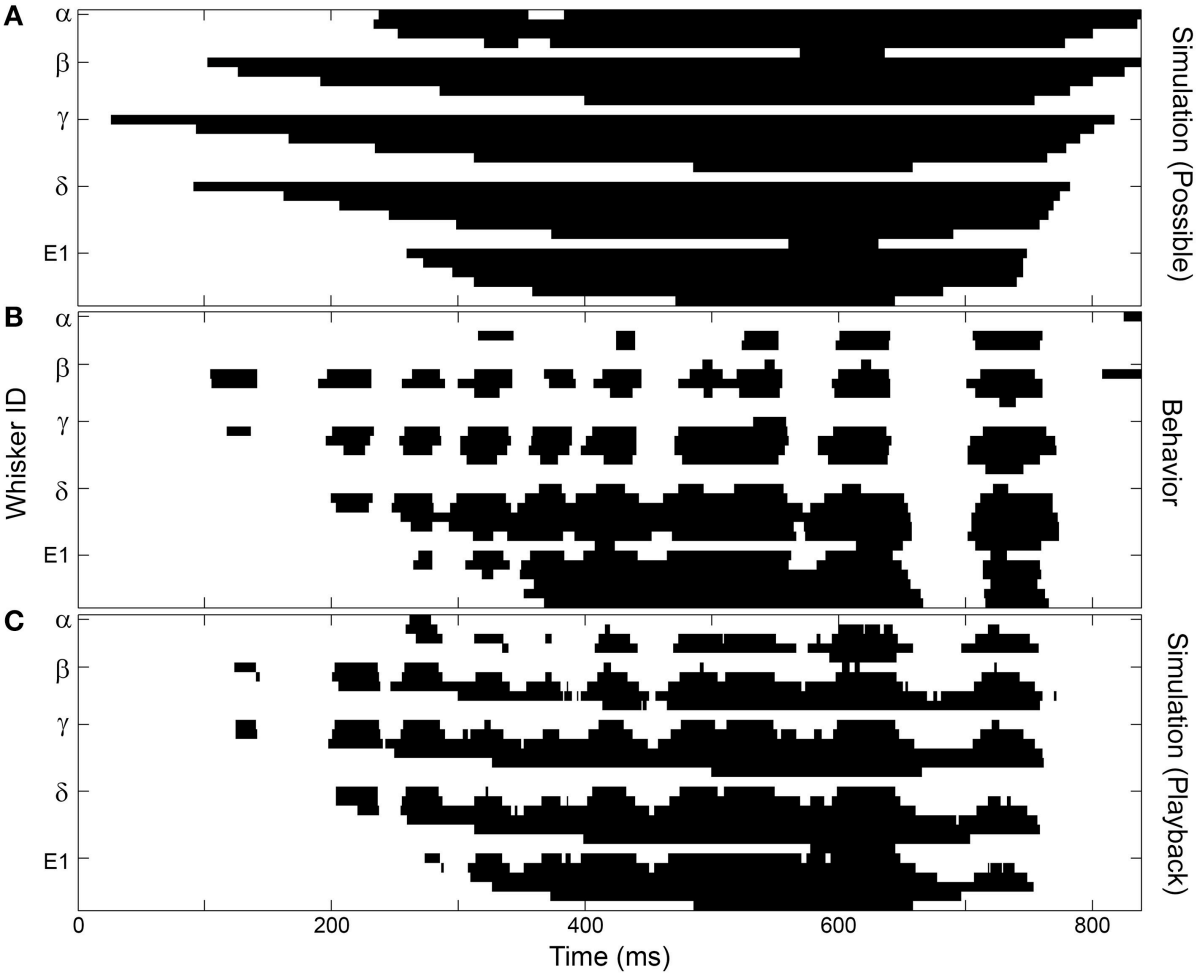
**The overall structure of whisker-surface contact can be captured by simulations that include head pose and average protraction angle**. This particular example is for the left array of Rat 3. Figure conventions follow those in Figure 5C. **(A)** A simulation that assumes maximal protraction at all head poses captures the overall spatial structure of contact, but none of the temporal features. **(B)** Behavioral data shows the characteristic SCCI structure. **(C)** Incorporating the measured protraction angles (along with the head position and orientation) reproduces much of the SCCI structure observed during natural behavior.

Results of the first simulation are shown in Figure 6A, and can be compared with behaviorally-measured contact patterns in Figure 6B. As expected, the simulation captures the overall spatial features of contact, showing gradual row-wise contact and detach, but completely lacks the discrete patterns of contact that so clearly characterize real behavior. The simulation shows that in almost every frame of the trial the vibrissae have the *potential* to make contact with the wall if they are protracted far enough forward, but this does not occur during real behavior.

Next, the effect of protraction angle was added to the simulations, in order to model the whisk cycle. In these simulations, the head was placed in the same position and orientation as the rat's head measured behaviorally at each point in time. Then, instead of simulating a complete protraction at each head pose, whiskers were placed at the same angle as the rostral- and caudal- most whiskers measured behaviorally. All other protraction angles were calculated by assuming that columns of whiskers were uniformly spaced between the rostral- and caudal- most whiskers (see Materials and Methods). Note that this simulation implicitly includes information about head velocity, because at each point in time the simulated head pose matched the head pose observed in the behavioral data. As shown in Figure 6C, these simulations recovered much, but not all, of the discrete structure seen in the observed behavior. The simulations did not—nor were they expected to—reproduce the exact identity of the whiskers that come into contact, nor the exact temporal structure of contact.

Instead, these results highlight the tight interplay between head pose, head velocity and the whisk cycle in determining contact patterns. Times of minimum head velocity provide regular temporal markers around which the SCCIs are centered and these times covary with the average peak protraction of the whisk cycle. The protraction angles in turn, are critical for the contact-detach patterns that produce the SCCIs. With this structure in mind, we next seek to further examine two key features of SCCIs: first, the exact contact/detach sequences at the edges of each SCCI, and second, the characteristics of whisker motion in the middle of each SCCI.

### Characterizing the sequences and timing of whisker contact and detach

#### Sequences of contact are equally likely to propagate in dorsal, ventral, rostral, or caudal directions

**Figure 7:**
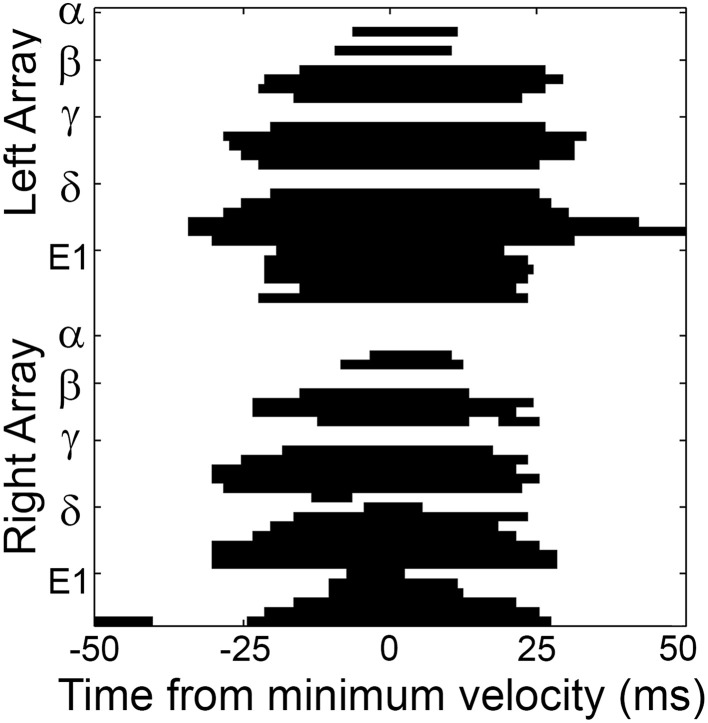
**A typical pattern of contact/detach is shown centered about the time of minimum head velocity (*t* = 0)**. Figure conventions follow those in Figure 5C. Whiskers are observed to “scatter on” to the surface, sustain contact within a temporal window around minimum head velocity (the SCCI), and then “scatter off” the surface. The exact sequence of whisker-object contact for this whisk is provided in the top half of Table 1.

With this understanding of the interaction between head pose and the whisk cycle, we next examined the precise sequence of contact/detach sequences that occurred around the “edges” of each SCCI. A typical example is shown in Figure 7. This example illustrates a well-isolated contact pattern in which almost every whisker is initially out of contact, maintains contact around the time of minimum head velocity (the SCCI), and then detaches. The whisker sequence of the contact pattern in Figure 7 is listed in the top half of Table 1. Using this approach the exact contact sequences were enumerated for all whisks for all rats at the 1 ms time scale.

**Table 1:** **Contact-detach sequences for the pattern of contact shown in Figure 7**.

**(Top)**	**Left Contact**	**Right Contact**	**Left Detach**	**Right Detach**
First	D4 D5	C3 C4 D4 D5 D6	A4	E6
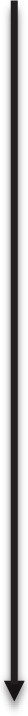	D6	C5	A2	C6
	C2 D3	C2	E1	E1
	C3	E6	E5	δ
	C4 D2	B2 B3 D3	B4	A2
	B3 C5 E6	E5	E2 E4 E6	E2
	B2 E2 E3 E4	D2	E3	A3 E3
	C1 D1	C1	C5 D1	B1 B4
	E1	D1 E4	B1 B3 C1	C1
	Time	B4	B1	D2
	B1 E5	C6	B2	B3 C3 D3 E4
	A4	B4	D3	C5
	A2	E2 E3	C3 C4 D6	C2 D1
		A3	C2	B2
		E1	D4	C4 D4 E5
		δ	[D5]	D5 D6
Last		A2		
**ROW-WISE**				
A Row	4-2	3-2	4-2	2-3
B Row	3-2-4-1	(2-3)-1-4	4-(1-3)-2	(1-4)-3-2
C Row	2-3-4-5-1	(3-4)-5-2-1-6	5-1-(3-4)-2	6-1-3-5-2-4
D Row	4-5-6-3-2-1	(4-5-6)-3-2-1-δ	1-2-3-6-4-5	δ-2-3-1-4-(5-6)
E Row	(2-3-4)-1-5	6-5-4-(3-2)-1	1-5-(2-4-6)-3	6-1-2-3-4-5

****(Top)*** The sequence of contact and detach for right and left sides of the array are listed. Vibrissae listed on the same row within a column of the table contact or detach simultaneously (to within 1 ms). The detach for Left D5 (in square brackets) occurs outside the time range shown in Figure 7, but is included as a detach because its contact was included. In contrast, Right E6 is in contact at the start of the time-series in Figure 7, but is not included as part of the contact/detach sequence because it was included with the sequence immediately prior. ***(Bottom)*** Row-wise: the sequences of contact and detach are shown for each row. Vibrissae that contact simultaneously (to within 1 ms) are enclosed in parentheses*.

Well-isolated contact sequences like that shown in Figure 7 were found for approximately two-thirds of the whisks. For the remaining third, defining the contact/detach sequence was complicated by contacts that were sustained over multiple whisks, and by whiskers that contacted and detached twice during a whisk. To include these cases, all sustained contacts were counted as the “first” contact in a sequence, and both instances of contact/detach were independently included in the sequence.

With the contact/detach sequence identified for each period of contact (corresponding approximately to a whisk), it was then possible to calculate the manner in which contact propagated over the entire array. One of the most prominent features of the data was the high level of variability between contact sequences. For example, the Levenshtein distance (“edit distance”) between any two sequences of contact was 88.1 ± 8.50% of the sequence length, meaning that any two sequences (chosen across all rats) shared only 11.9 ± 8.50% of the same (ordered) contacts. This distribution also held for sequences of vibrissal-object contact generated by sequential whisks (*p* = 1, Wilcoxon rank-sum test). Furthermore, for each rat considered individually, the Levenshtein distance between sequences of contact was not significantly different from the Levenshtein distance between any two sequences of contact chosen across rats. The within-rat Levenshtein distances between sequences of contact were: Rat 1: 85.12 ± 7.67%; Rat 2: 88.63 ± 11.20%; Rat 3: 91.26 ± 7.05%; Rat 4: 79.90 ± 6.12%; Rat 5: 85.43 ± 9.90%.

**Figure 8:**
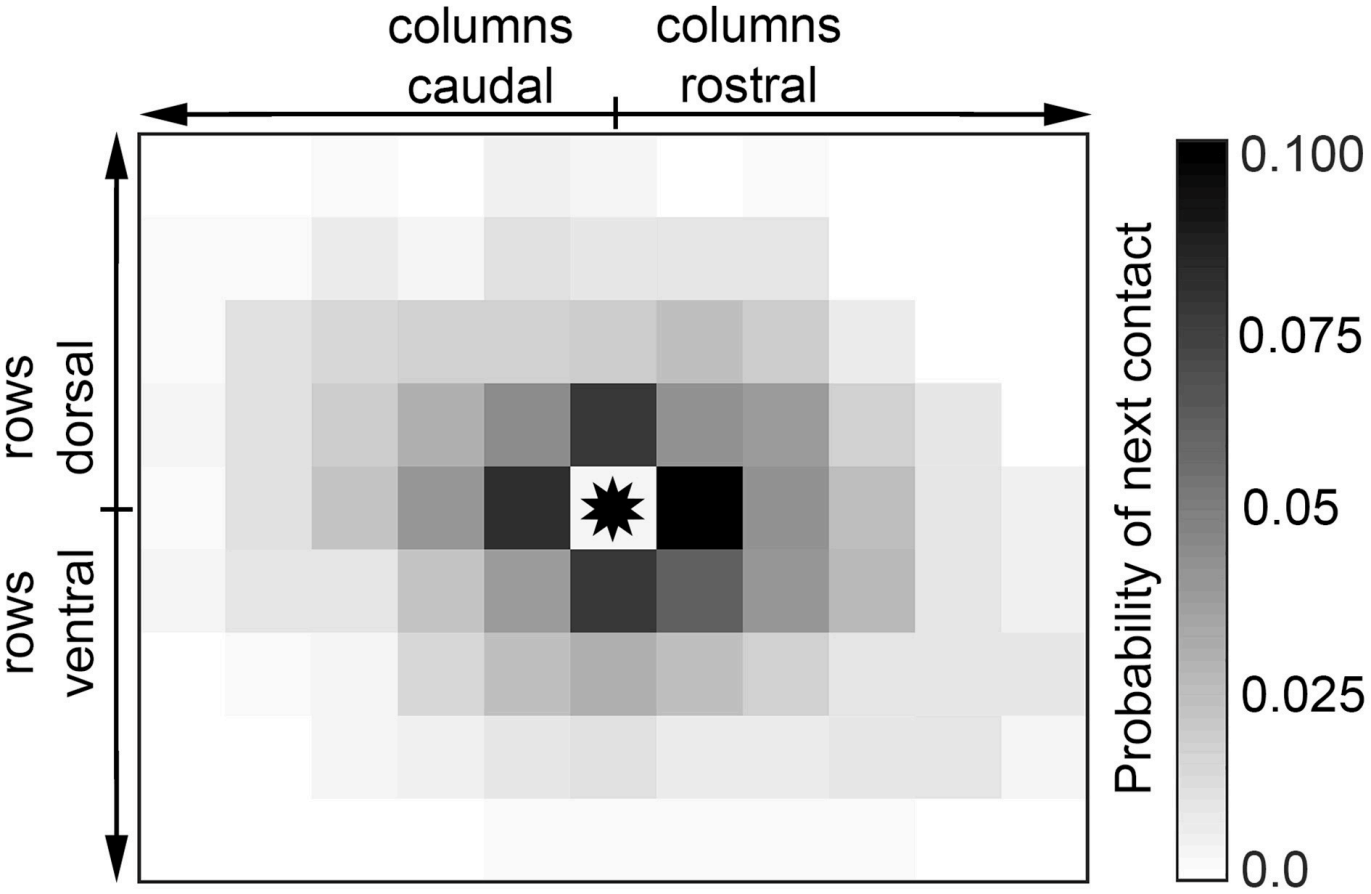
**Contact is equally likely to propagate to any direct nearest neighbor**. Given that a particular vibrissa is in contact (asterisk in the center), propagation is equally likely to continue in the direction of any of its four nearest neighbors.

The high variability in contact sequences was also evident upon examination of the detailed structure of sequential contacts. Figure 8 shows the probability distribution of sequential contacts: given a whisker in contact, the probability that each other whisker will contact next is shown. The figure reveals that when a whisker contacts the glass, each of its direct nearest neighbors is equally likely to contact next (*p* = 0.16, not significantly different from uniform, χ^2^ test). Similarly, the direction of propagation is equally likely among its four diagonal nearest neighbors (*p* = 0.19, not significantly different from uniform, χ^2^ test). However, a direct nearest neighbor is more likely to follow a contact than a diagonal nearest neighbor (*p* = 1.8E^−5,^ χ^2^ test).

#### Sequences or contact are somewhat structured within a row; column 2 vibrissae are often the first to make contact

Given the well-known row-wise receptive field structure of many central neurons in the vibrissal-trigeminal system (Simons, 1978, 1985; Jacquin et al., 1989; Chakrabarti and Alloway, 2009; Lustig et al., 2013; Ramirez et al., 2014), we next aimed to quantify sequences of contact within single rows. The bottom half of Table 1 provides an example of the approach taken to identify sequences within a row based on the example contact pattern shown in Figure 7. Sequences were identified for the right and left sides of the array independently, across all 10 rows (five on each side of the face).

Using this approach, row-wise contact sequences were quantified for the 77 distinct contact periods for the 10 rows of whiskers. Of the 770 total row sequences in the dataset, 568 had at least one whisker in contact, while 202 sequences were “empty,” meaning that no whiskers within that row made contact. The large number of empty sequences is not a concern; it is common for numerous whiskers to be in contact across the array while an entire row remains out of contact. This situation often occurs when the head is pitched strongly upward (leaving the dorsal whiskers out of contact), strongly downward (leaving the ventral whiskers out of contact), or to the side (sometimes leaving all five rows on the more distant side of the face out of contact).

The 15 most common row-sequences, including those “sequences” in which only a single whisker in the row made contact, are listed in Table 2A. These 15 row-sequences comprise nearly 40% (224 out of 568, or 39.4%) of all row-sequences. In this analysis, sequence subsets are considered distinct. For example, the sequence 2-1-3 with no other whiskers in contact is considered distinct from the sequence 2-1-3-G. Unsurprisingly, therefore, the sequences in Table 2A tend to be short, because sequences involving larger numbers of whiskers permit more variability.

**Table 2:** **The statistics of contact sequences within a row**.

**A**	
**Row sequence**	**Number of occurrences**
1	34
2-1	29
2	27
2-1-3	25
2-3-1	19
1-2	15
G	12
3	11
3-1	8
3-2-1	8
3-2	8
2-1-G	7
2-1-3-G	7
2-3	7
(2-1)	7
All other row-sequences occurred fewer than seven times	
**B**	
**3-whisker Start sequence**	**Number of occurrences**
2-1-3	47
2-3-1	32
3-2-1	22
2-1-G	11
3-4-2	9
4-3-2	7
(3,2)-1	8
(3,2)-4	8
2-3-4	6
3-2-4	6
All other 3-whisker start-sequences occurred fewer than six times	

***(A)** The 15 most common row-sequences and the number of times they occurred are listed. The letter G indicates the Greek column. Sequences are treated as distinct even if they are a subset of another sequence; for example, 2-1-3 is distinct from 2-1-3-G. Whiskers in parenthesis indicate contacts simultaneous to within 1 ms. ***(B)*** The 10 most common 3-whisker start sequences and the number of occurrences are listed. Row-sequences with fewer than three contacts are excluded from this analysis. Whiskers in parenthesis indicate contacts simultaneous to within 1 ms*.

To interpret the results shown in Table 2A, it is helpful to “walk through” a few rows of the table. The first row of the table indicates that 34 times, a Column 1 whisker made contact with the surface, but no other whiskers in that same row of whiskers made contact. The second row of Table 2A indicates that 29 times, a Column 2 whisker made contact, followed by a Column 1 whisker in that same row of whiskers. When considered over all row-sequences, one of the most striking features of Table 2A is the complete absence of whiskers from columns 4, 5, and 6. Even though the nose was almost always close enough to bring these rostral whiskers into contact, they were very rarely the first whiskers to contact the surface.

As noted previously, Table 2A tabulates sequences of contact while distinguishing between sequence subsets. An alternative approach, shown in Table 2B, is to uniquely identify sequences based on the initial set of whiskers that make contact. Specifically, Table 2B quantifies the statistics of the first three whiskers to make contact with the glass within a row (“3-whisker start sequences”). In this analysis, two sequences are counted as the “same” as long as their first three contacts are ordered in the same way. For example, the start sequence 2-3-1 includes contact sequences 2-3-1, 2-3-1-G, and 2-3-1-5-4, as well as any other contact sequence that starts with 2-3-1. The results of Table 2B show that the three most probable start-sequences involve only whiskers from Columns 1, 2, and 3. Together, these top three sequences started over a quarter of the row-sequences (101 out of 568, or 27.4%). None of the top 10 start-sequences involve the rostral most whiskers from Columns 5 or 6, but whiskers from Column 4 appear in half of the top 10 start-sequences.

**Figure 9:**
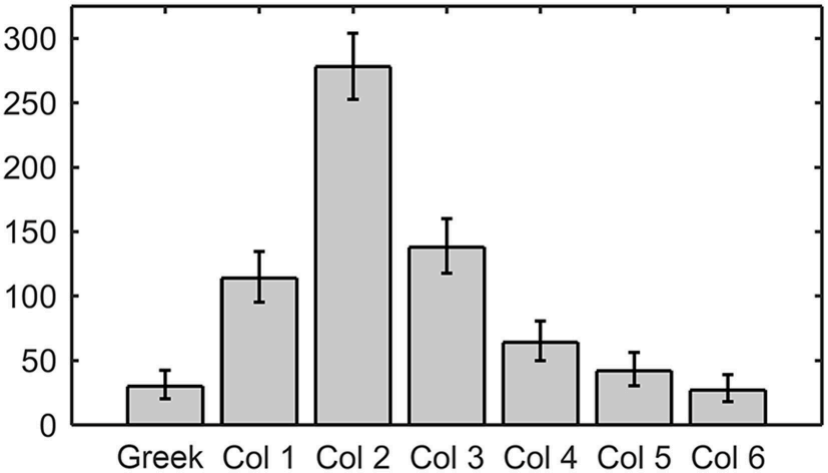
**Column 2 whiskers are by far the most likely to contact first within their row**. Error bars correspond to the 95% confidence interval.

When examined together, Tables 2A,B show that a disproportionate number of row-sequences begin with contact by a Column 2 whisker. This result is further quantified in Figure 9, which illustrates the number of times that whiskers of a given column were the first to contact within the row. Column 2 vibrissae are almost twice as likely to contact first within a row than whiskers in any other column (*p* < < 0.001 compared to uniform).

Finally, to generalize results of this row-wise analysis, the 3-whisker start sequences were classified into four types based on the direction of propagation within a row: rostral-caudal (RC), caudal-rostral (CR), center-out (CO), and irregular (IRR). Definitions of these sequence types and the frequency with which they occurred (as a percentage of the total number of row sequences) are shown in Table 3.

**Table 3:** **Row-wise contact sequences**.

**Pattern type**	**Definition (assuming three contacts)**	**Observed percentage**	**Theoretical percentage**
Rostral-Caudal (RC)	**21** sequences meet the RC definition: A row (3): 4-3-2, 3-2-1, 2-1-α B row (4): 5-4-3, 4-3-2, 3-2-1, 2-1-β C row (5): 6-5-4, 5-4-3, 4-3-2, 3-2-1, 2-1-γ D row (5): 6-5-4, 5-4-3, 4-3-2, 3-2-1, 2-1-δ E row (4): 6-5-4, 5-4-3, 4-3-2, 3-2-1	67/568 = 11.80%	21/720 = 2.92%
Caudal-Rostral (CR)	**21** sequences meet the CR definition: A row (3): α-1-2, 1-2-3, 2-3-4 B row (4): β-1-2, 1-2-3, 2-3-4, 3-4-5 C row (5): γ-1-2, 1-2-3, 2-3-4, 3-4-5, 4-5-6 D row (5): δ-1-2, 1-2-3, 2-3-4, 3-4-5, 4-5-6 E row (4): 1-2-3, 2-3-4, 3-4-5, 4-5-6	18/568 = 3.17%	21/720 = 2.92%
Center-Out (CO)	**42** sequences meet the CO definition: A row (6): 1-α-2, 1-2-α, 2-1-3, 2-3-1, 3-4-2, 3-2-4 B row (8): 1-β-2, 1-2-β, 2-1-3, 2-3-1, 3-4-2, 3-2-4, 4-3-5, 4-5-3 C row (10): 1-γ-2, 1-2-γ, 2-1-3, 2-3-1, 3-4-2, 3-2-4, 4-3-5, 4-5-3, 5-4-6, 5-6-4 D row (10): 1-δ-2, 1-2-δ, 2-1-3, 2-3-1, 3-4-2, 3-2-4, 4-3-5, 4-5-3, 5-4-6, 5-6-4 E row (8): 2-1-3, 2-3-1, 3-4-2, 3-2-4, 4-3-5, 4-5-3, 5-4-6, 5-6-4	160/568 = 28.17%	42/720 = 5.83%
Irregular (IRR)	**636** sequences are identified as IRR because they are not RC, CR, or CO: A row: 60 − (3+3+6) = 48 B row: 120 − (4+4+8) = 104 C row: 210 − (5+5+10) = 190 D row: 210 − (5+5+10) = 190 E row: 120 − (4+4+8) = 104 48+104+190+190+104 = **636**	323/568 = 56.90%	636/720 = 88.33%

*Definitions of rostral-caudal (RC), caudal-rostral (CR), center-out (CO), and irregular (IRR) start sequences. The percentage of the time that these sequences were observed experimentally is shown in column 3, and can be compared with the percentage of the time that they would have been observed if all sequences were equally likely (column 4). The calculations assume that the A row has five whiskers, the B and E rows both have six whiskers, and the C and D rows both have seven whiskers*.

The experimentally-observed percentages of occurrence (column 3 of Table 3) were then compared with the percentage with which they would have theoretically been observed if all contact sequences were equally likely (column 4 of Table 3). The theoretical percentage for each type (e.g., RC) is calculated as the number of patterns in that type divided by the total number of possible sequences. The theoretical calculations assume that the A row has five whiskers, the B and E rows both have six whiskers, and the C and D rows both have seven whiskers (Knutsen et al., 2008; Towal et al., 2011). Notably, the CO and RC sequences occur far more frequently than their theoretically predicted frequencies (*p* < 0.001).

Summarizing, the results of Figures 7–9 and Tables 1–3 provide evidence that when considered across the entire array, sequences are equally likely to propagate in all four cardinal directions. When considered on a row-wise basis, contact sequences exhibit somewhat more structure, with column 2 whiskers tending to make contact first, and with CO and RC sequences more probable than would be predicted if all contact sequences were equally likely.

#### Contact timing

**Figure 10:**
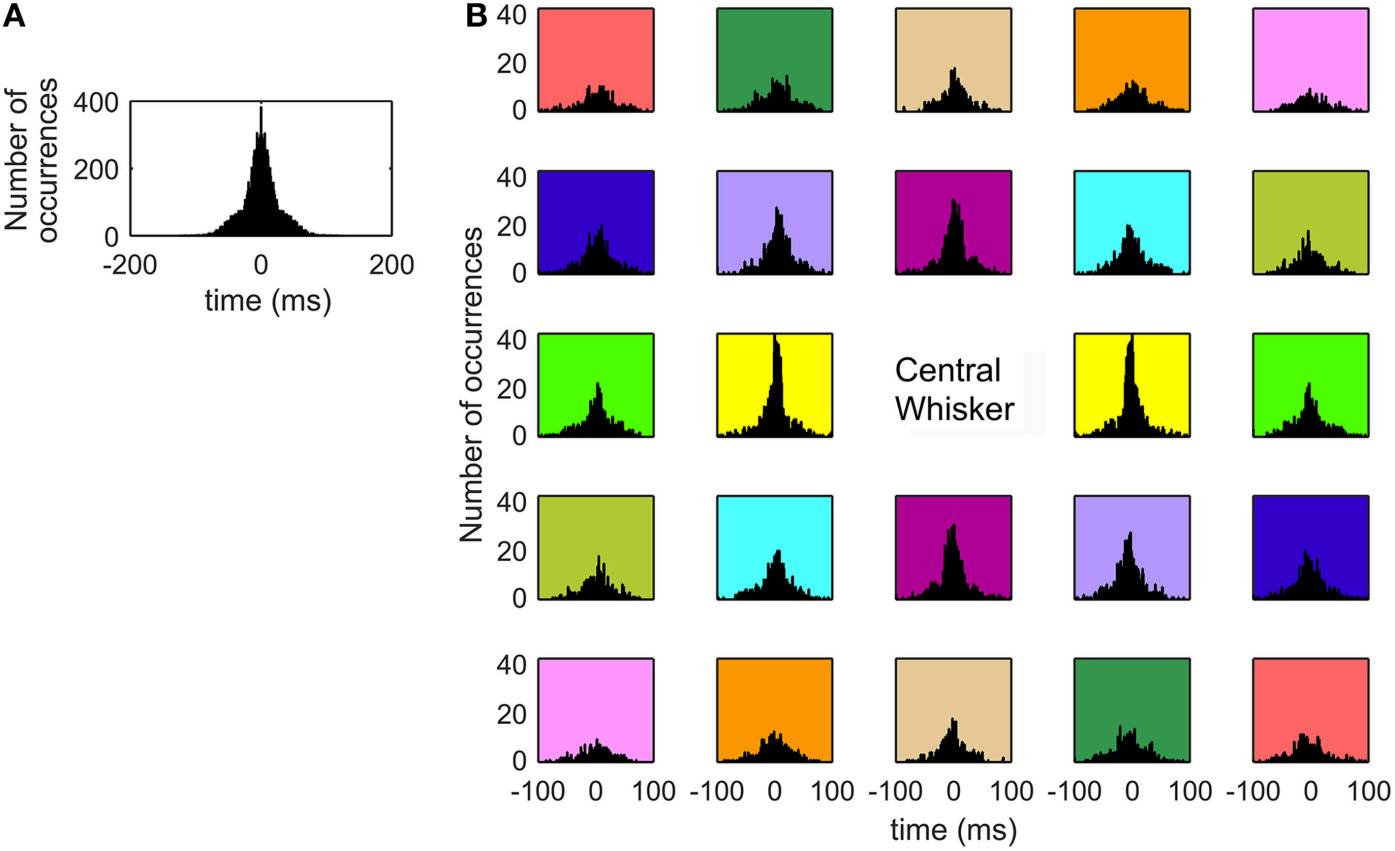
**Inter-whisker contact intervals exhibit a strong central peak. (A)** The histogram shows the distribution of time differences between when one whisker made contact with the surface and when all other whiskers in the same row made contact with the surface. Approximately 71% of the distribution's mass falls within ±25 ms. **(B)** Inter-whisker contact intervals between a whisker and each of its neighbors, averaged over all whiskers. The eight immediate neighbors of the center whisker exhibit strong central peaks, while whiskers more distant from the center whisker show broader distributions of inter-whisker contact intervals. The subplots are colored in a manner intended to help visualize the “symmetry” about the central whisker. Each pair of subplots with matching background color shows histograms that are reflections of each other about the y-axis.

It is well-established that inter-whisker contact timing will have a strong influence on the neural responses generated at many levels of the trigeminal system (Benison et al., 2006; Rodgers et al., 2006; Simons et al., 2007; Khatri et al., 2009b; Ramirez et al., 2014), and we therefore next examined the inter-whisker contact intervals for whiskers within a row. Figure 10A shows the distribution of time differences between when a whisker made contact and when every subsequent whisker in that row made contact. For example, if C2 contacts at *t* = 10 ms, C3 at *t* = 15 ms, C4 at *t* = 16 ms, and C5 at *t* = 18 ms, the timing intervals will be ±5, ±6, ±8, ±1, ±3, and ±2 ms. The distribution is (by definition) symmetric about zero. It has a standard deviation of 27.79 ms. It exhibits a sharp central peak within approximately ±25 ms and then broadens dramatically. 70.98% of the distribution's mass falls within ±25 ms.

To characterize timing differences across the array, inter-whisker contact intervals between a whisker and all of its neighbors are plotted in Figure 10B, averaged over all whiskers. The subplots in this figure have been color coded to emphasize a form of symmetry about the central whisker. Each of the subplots with the same background color show histograms that are reflections of each other about the y-axis. The reason for the symmetry is that if Whisker A is 2 caudal and 1 dorsal from Whisker B, then Whisker B is 2 rostral and 1 ventral from Whisker A. The inter-whisker contact intervals are therefore simply negatives of each other.

The histograms of Figure 10B reveal that the inter-whisker contact intervals for the eight neighboring whiskers that immediately surround the center whisker all show large and narrow central peaks. In contrast, whiskers that are more distant from the center whisker are associated with histograms with broader distributions and much weaker central peaks. These more distant whiskers often contact up to 80 or 90 ms after the center whisker.

### Whiskers decrease speed and spatially converge on the object during the intervals of sustained, collective contact

The previous section has characterized the sequences and temporal structure of the contact and detaches that form the “edges” of each pattern. This section now characterizes what happens to the whiskers *during* the SCCI, that is, *during* the windows of sustained contact with the vertical surface.

**Figure 11:**
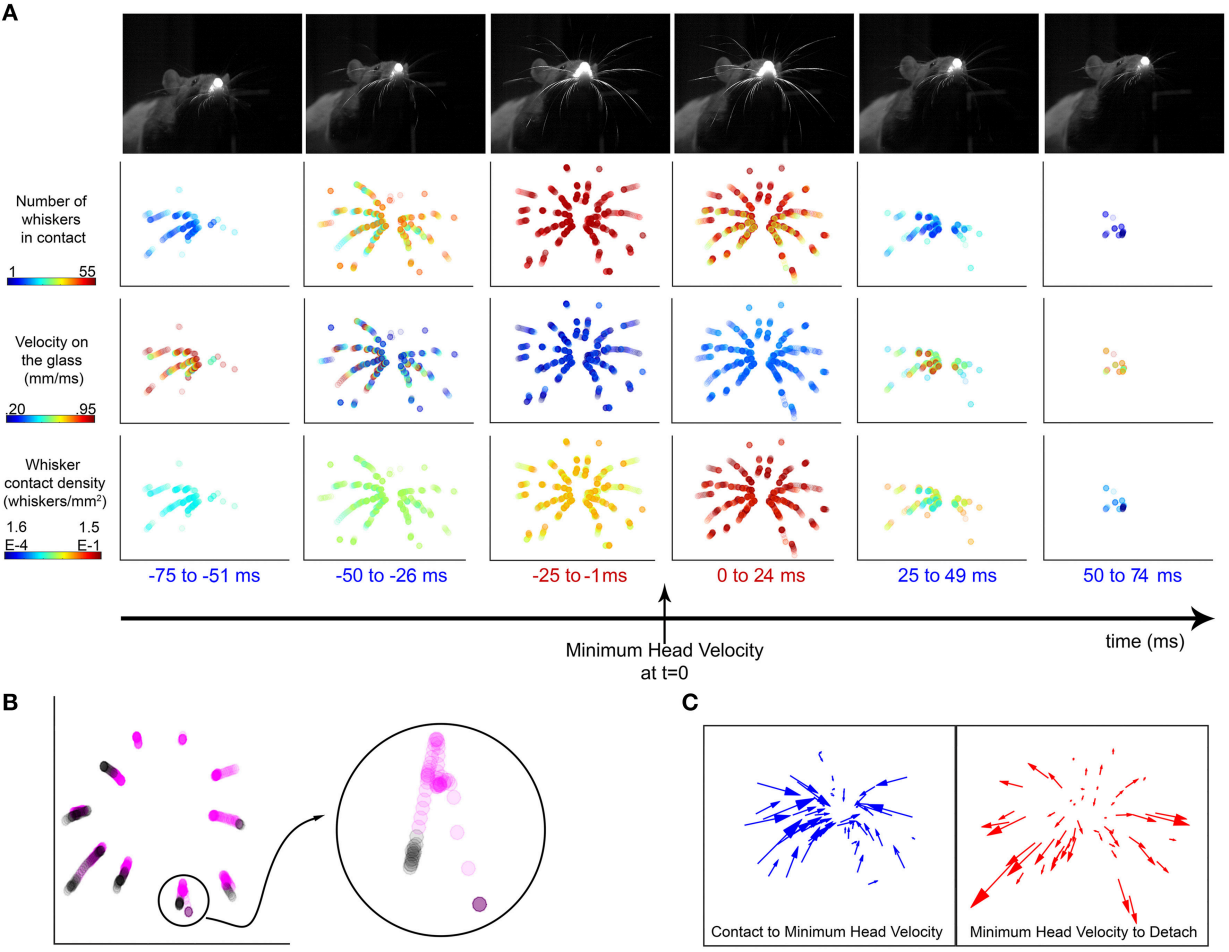
**Vibrissal-surface contact points slow down and converge around times of minimum head velocity. (A)** An example whisk illustrates the number, velocity, and density of whiskers on the surface around the time of minimum head velocity. Top row: Each image of the rat is from a single video frame taken near the middle of the time window indicated. The images are from the head-on camera view, taken through the glass pane. Rows 2–4: In each panel, the dots indicate whisker-surface contact locations during the specified time window, all superposed into a single image. Each dot represents the location of one whisker-surface contact point. Because each time window lasts 25 ms and the rat has 62 whiskers (31 right and 31 left), the maximum number of dots in any panel is 1550. Row 2: The number of whiskers in contact with the glass is maximum during the 25 ms before and after the time of minimum head velocity. The color of each dot represents the number of whiskers in contact with the surface in that frame, so all dots from the same video frame have the same color. Row 3: The velocities of the contacts are minimized during the 25 ms before and after the time of minimum head velocity. The color of each dot represents the velocity of the whisker at that contact point location. Row 4: The density of whiskers is maximum during the 25 ms before and after the time of minimum head velocity, as the whiskers converge on the sheet. The color of each dot represents the density of whiskers in contact with the surface in that frame, so all dots from the same video frame have the same color. For each video frame, density was computed as the number of whiskers in contact with the surface divided by the area of the convex hull of the whisker contacts. **(B)** The trajectories of the Column 2 vibrissae are plotted in the ±75 ms interval around minimum head velocity. Points within ±25 ms of minimum head velocity are colored magenta and points outside that window are colored black. The whiskers contact the glass, move inward toward the nose, and then move away from the nose before detach. (Inset) The trajectory of the E2 vibrissa is enlarged for clarity. Its trajectory shows a “loop” signature, characteristic of almost every contact for all whiskers, all rats. **(C)** (Left) The trajectory of each whisker is represented as a vector from its location at contact to its location at minimum head velocity. The overwhelming majority of whisker contact locations move inwards, toward the nose. (Right) The trajectory of each whisker is represented as a vector from its location at minimum head velocity to its location of detach. After minimum head velocity, almost every whisker moves outwards, away from the nose before detaching.

Figure 11A shows the locations of contact on the glass from −75 ms prior to +75 ms after the time of minimum head velocity of a representative whisk. The example shows that as the head approaches minimum velocity, the number of whiskers on the surface increases. At the same time the speed of the contact points decreases, and the density of contacts increases (i.e., the contact points converge).

What these results mean physically is that as the rat increasingly deflects its whiskers against the surface, the whiskers bend. The contact points on the whiskers become increasingly proximal. This convergence, with the contact points moving closer in toward the nose is also clearly visible in Movie 1.

This convergence is also evident when plotting the trajectories of a single column of whisker contact locations, as seen in Figure 11B. The contact locations 25 ms before and after minimum head velocity are colored magenta, while contact locations outside of this window are colored black. By examining a single whisker (E2, seen in the inset), a clear “loop” structure is visible; the whisker moves toward the nose during the first part of its trajectory, then turns and moves back outward. This loop was observed for all whiskers during all whisks for all trials for all rats, except in cases where contact and detach both happened on the same side of peak protraction. Factors that may contribute to the loop structure are head movements, the roll and elevation of the whisker's kinematic trajectory, and frictional interactions between the whisker and glass surface.

This convergence effect can be further highlighted by plotting the trajectory vectors of the whiskers from the time of contact to the time of minimum head velocity, and from minimum head velocity to detach (Figure 11C). Nearly all whiskers exhibit a trajectory inward toward the nose from the time they contact until minimum head velocity; then reverse to move back outward away from the nose.

**Figure 12:**
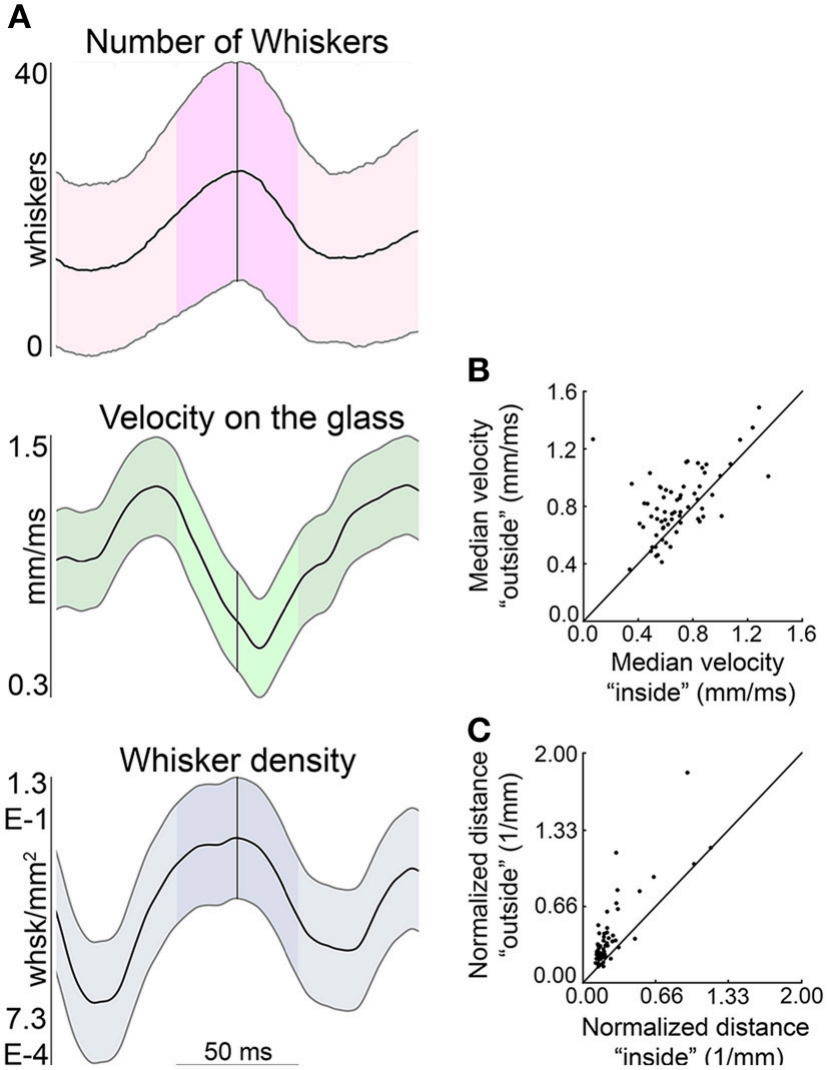
**The contact points of the whiskers on the glass decrease speed and converge near times of minimum head velocity. (A)** The average number of whiskers in contact, velocity on the glass, and whisker density are each centered about the times of minimum head velocity (across all whisks of all rats). The time of minimum head velocity is indicated by the vertical gray line and ±25 ms window is highlighted in each plot. The mean in each subplot is shown as a thick black line, with standard deviation as thinner black lines above and below the mean. **(B)** The velocity of the contact location on the glass ±25 ms around the time of minimum head velocity is signficantly smaller than outside that time interval. **(C)** The distance between contact locations is significantly smaller in the ±25 ms around minimum head velocity than outside that time interval.

This analysis is extended across all whisks of all rats in Figure 12. Figure 12A shows that near times of minimum head velocity the average number of whiskers in contact is maximum, the contact point velocity on the glass is near minimum, and whisker density is near maximum. Figure 12B illustrates that the velocity of the contact locations is reduced within the ± 25 ms surrounding minimum head velocity (*p* < 0.025). Additionally, the distance between vibrissal contacts on the glass (normalized by the contact area as given by the convex hull of the contact points) is significantly smaller during this same time period (*p* < 0.005), as seen in Figure 12C. All results were robust to varying the time window between ±15 and ± 35 ms. Thus, the whiskers can be seen to slow down and converge, effectively increasing sampling resolution.

## Discussion

The present study is the first to characterize patterns of vibrissal-object contact across the entire vibrissal array during natural tactile exploration. All head movements and whisker contacts were quantified, both spatially and temporally, over multiple whisks with 1 ms resolution. The light sheet technology also permitted quantification of how whiskers move during contact with the surface, both individually and relative to other whiskers in the array.

### Three spatiotemporal scales for control of vibrissotactile data acquisition

The present work adds to a growing body of evidence that head movements are critical in determining how the vibrissae are brought into contact with a surface of interest (Catania and Remple, 2004; Mitchinson et al., 2007; Grant et al., 2009; Huet and Hartmann, 2014; Hobbs et al., 2015,in press). As suggested by previous studies (Hobbs et al., in press) and shown in Figure 6A, the *possibility* of contact is determined almost entirely by head pose and array geometry. Whether a specific vibrissa then actually makes contact with the surface is largely shaped by the whisk cycle. Finally, the velocities of individual vibrissae ultimately determine the exact contact sequence. Thus, there are at least three scales over which the rat can potentially control sensory data acquisition: at the level of the head, at the level of the whisk cycle, and at the level of individual vibrissal motion.

The present work also adds to overwhelming evidence that the rat has a high degree of differential control between groups of whiskers (Berg and Kleinfeld, 2003; Hill et al., 2008; Knutsen et al., 2008; Haidarliu et al., 2010, 2013) During natural behavior, the whisk cycle is often extremely complex, exhibiting characteristics such as delayed and double pumps, right/left asymmetries, changes in spread, and significant phase differences between whiskers in the rostral and caudal regions of the array (Towal and Hartmann, 2006, 2008; Mitchinson et al., 2007; Grant et al., 2009). All of these complexities are observed even during non-contact whisking (Towal and Hartmann, 2006, 2008), and only increase once the whiskers contact an object (Mitchinson et al., 2007; Grant et al., 2009; Deutsch et al., 2012).

Although the whisk cycle is clearly quasi-periodic, the complexity of whisking kinematics during natural behavior precludes the meaningful identification of a single time of protraction/retraction or a single “protraction angle” across the array. Unlike in the head-fixed rat, the whisk cycle is not uniquely defined when the rat is free to move and explore. It is possible, however, to lock analysis on periodic fluctuations in head velocity (Welker, 1964; Hartmann, 2001; Catania and Remple, 2004; Grant et al., 2009), the minima of which might be likened to visual fixations (Catania and Remple, 2004). The “loosely locked” nature of the head velocity and whisk cycle allows us then to examine the spatiotemporal structure of contacts around times of minimum head velocity.

### Combined head and whisker movements lead to complex sequences of contact

Given the complex interplay between head and whisker movements it is not surprising that contact and detach sequences of the vibrissa on the surface are highly complex and varied as well. Free exploration enables the rat to explore the surface in a variety of poses and thus generates a wide range of contact patterns and sequences (Sellien et al., 2005; Hobbs et al., in press). Figure 8 and the Levenshtein distance analysis of the present work demonstrate that when considered across the array as a whole, sequences are equally likely to propagate in any direction across the array, and that any two sequences shared < 12% of the same (ordered) contacts. This degree of variability is present both within and between rats.

More structure is evident when sequences of contact/detach are analyzed on a row-wise basis (bottom half of Table 1 as well as Tables 2, 3). Intuition might suggest that vibrissal protraction against a rostrally-located surface will generate a rostral-caudal sequence of vibrissae-object contact followed by a caudal-rostral detach sequence during retraction. However, this combination of contact/detach pattern was observed < 12% of the time (Table 3). Instead, center-out contact sequences were found to be the most common (28%). The prevalence of CO sequences is not unanticipated, as work in simulation has shown that the central vibrissae are often in contact with a vertical surface in front of the nose even when the rostral vibrissae are not (Hobbs et al., 2015,in press). Furthermore, CO contact patterns are potentially consistent with the center-surround receptive fields present at many stages of the vibrissal-trigeminal pathway.

### Spatiotemporal contact patterns exhibit a temporal window in which the whiskers sustain contact with the surface

In contrast to the tremendous variability found to characterize contact and detach sequences, a feature that characterized 100% of whisks for all rats was a brief interval of sustained, collective vibrissal-object contact, which we term the SCCI. Across all whisks of all rats, the majority of whiskers remained on the glass for durations longer than 40 ms. During the SCCI, the whiskers continue to protract against the surface; the speed of the vibrissal contact points on the surface decreases, and the contact points move closer together, converging toward the nose as they slip more proximally along the whisker (Figures 11, 12).

Importantly, these are exactly the mechanical conditions required to damp out vibrations associated with the whiskers' initial collision with the surface (Boubenec et al., 2012; Yan et al., 2013; Quist et al., 2014). In a previous simulation study (Quist et al., 2014), we showed that the mechanical signals generated during one whisk will typically interfere with those generated by the next. Because vibrissal dynamic effects take at least 20 ms to damp out, it was predicted that during tactile exploration, rats will “press in” their vibrissae against surfaces for durations between 20 and 60 ms, so as to damp the dynamic response. These extended contact durations have already been observed in some studies (Deutsch et al., 2012). Because the vibrissae are highly damped (Hartmann et al., 2003; Neimark et al., 2003) vibrations will either die out during this sustained contact, or will be related to the microtexture or friction of the object (Ritt et al., 2008; Wolfe et al., 2008).

We have deliberately avoided assigning a specific duration to the SCCI because it varies considerably between whisks. The analyses in the present work were performed using a ±25 ms window around times of minimum head velocity, but all results were confirmed to hold equally well using windows between ±15 and ±35 ms. Windows smaller than ±15 ms do not adequately capture the sustained nature of the contacts, while windows larger than ±35 capture too large a fraction of the whisk cycle. The value of ±25 ms was chosen in part because Figure 5A shows that on average, the number of whiskers in contact with the glass reaches about two-thirds of its maximum value 25 ms on either side of minimum head velocity.

Finally, the prominence of the SCCI may speak to the motor control strategies that the rat may use during different phases of tactile exploration. During non-contact whisking and object localization, the rat could use a position control strategy, because no significant external forces are acting on the whisker-follicle complex, and because the whiskers are light enough to permit position control (Quist et al., 2014). In contrast, once contact is made with the object, the rat may transition to a force control strategy, as contact forces become significant and begin to aid in the determination of the 3D location of vibrissal-object contact (Kaneko et al., 1998; Solomon and Hartmann, 2011; Bagdasarian et al., 2013; O'Connor et al., 2013; Pammer et al., 2013; Huet et al., 2015). We note that analogous ideas for this type of position-force transition are largely uncontroversial in the literature on human reaching and grasping (Salisbury and Craig, 1982; Xiao et al., 2000; Robles-De-La-Torre and Hayward, 2001).

Our view, then, is that rat contact-whisking behavior may be thought of almost like a grasp, with the whiskers encircling the object and performing a haptic procedure that resembles “enclosure” (Klatzky and Lederman, 1995; Lederman and Klatzky, 2009).

It will be important for future studies to assess how the present results generalize to the rat's exploration of non-flat surfaces. Some initial results from simulation studies (Hobbs et al., 2015) indicate that vibrissal-surface contact sequences will become even more variable if the surface is curved or irregular. If, as we suggest, the SCCI is critical to the rat's determination of object features, then we specifically predict that the rat will adjust its head pose and whisking speed so as to maintain this feature of whisking behavior.

### A vibrissal haptic glance

The present work deliberately employed a task in which the rat explored a novel object, and did not perform a tactile discrimination. This type of “novelty induced” whisking surpasses even discriminative whisking as measured by number of whisks, whisk amplitude, and whisk velocity (Harvey et al., 2001; Berg and Kleinfeld, 2003; Wu et al., 2013), and is most likely to reveal the patterns of vibrissa-object contact characteristic to rapid haptic exploration; these patterns will be masked during vibrissa-mediated discriminative tasks in which the animal learns to extract a single salient cue in the shortest possible time.

Our choice of task was thus motivated by the understanding that during exploration of an unfamiliar object, the rat must obtain a reliable, if coarse, approximation of the object's location and contours within a few whisks (Knutsen et al., 2008; Horev et al., 2011; Saig et al., 2012; Yu et al., 2013; Arkley et al., 2014). In other words, the rat must perform a “haptic glance” (Klatzky and Lederman, 1995; Lederman and Klatzky, 2009) with its vibrissae. The spatial information that the rat obtains during this glance must be rapid, reliable, and robust to variations in head pose and whisking velocity. The present study, however, finds that the times and sequences of vibrissae-object contact are highly variable and that the most consistent feature of the tactile exploratory behavior is the interval of sustained vibrissal-object contact.

How then, might the rat obtain reliable information about the spatial features of objects within 1–3 whisks? We consider the plausibility of two possible coding schemes to answer to this question, while emphasizing that we do not claim to offer definitive proof in favor of either scheme.

The first possibility is that the rat could keep track of head motion, precise inter-vibrissal timing, and the precise sequences of contact to aid in the determination of the spatial features of the object. This type of strategy could be useful in situations when the rat is able use quite stereotyped head and vibrissal movements, for example during the over-trained tactile discrimination of a single, well-identified salient cue.

Although such an exploratory strategy is certainly possible, the present data suggest to us that it is extremely unlikely. In order to make use of timing information, the rat would have to normalize the incoming sensory data for head position and orientation, and keep track of vibrissal velocity to a resolution not observed in responses of the primary sensory neurons of the trigeminal ganglion (Leiser and Moxon, 2006, 2007; Khatri et al., 2009a). Efference copy could potentially provide a copy of the velocity command signal, but the rat would have no peripheral “handshake” that the commanded velocity was actually obtained. The problem is further complicated because a rat's vibrissae change length on a day-to-day basis from barbering, damage, and regrowth.

As an alternative scheme, we offer the following “windowed sampling” hypothesis: the rat does not rely primarily on inter-vibrissal timing cues to extract the object's spatial features, but instead spatially integrates mechanical signals across whiskers acquired during the brief window of sustained vibrissal contact (the SCCI).

This hypothesis leads to an overall model of vibrisso-haptic exploration in which temporal cues are critical for object localization (Ahissar and Arieli, 2001; Knutsen and Ahissar, 2009) and for texture determination (Arabzadeh et al., 2003, 2005; Lottem and Azouz, 2008, 2009; Ritt et al., 2008; Wolfe et al., 2008; Jadhav and Feldman, 2010), but the spatial features of an object (e.g., curvature) are estimated by integrating mechanical information across vibrissae during a brief window of quasi-static deflection that lasts only a fraction of the whisk.

The strategy suggested by the windowed sampling hypothesis offers at least three compelling advantages over a scheme that depends on precise temporal intervals of inter-vibrissae contact.

First, integrating spatial information within a single temporal window eliminates the variability in inter-vibrissae-timing arising from variations in head and whisking movements. The rat could thus obtain an initial tactual impression of the object within a single whisk, regardless of the precise temporal structure (sequence) with which the vibrissae happen to make contact with the object.

Second, the strategy does not require memory, as would be required for integration and inter-vibrissal comparisons across time.

Third, if the rat's perception of object contours does not depend on the temporal structure of sensor contacts, then variations that do occur at that timescale could be used to detect object motion, compliance (Kaneko et al., 1998; Pammer et al., 2013), or texture (c.f., Kepecs et al., 2006).

Windowed sampling as described here resembles the periodic sampling strategy associated with sniffing and visual saccadic behavior (Yarbus, 1967; Kepecs et al., 2006), as well as the separation of encoding and retrieval of memories during different phases of the hippocampal theta rhythm (Hasselmo et al., 2002). Finally, the SCCI described in the present study could potentially represent a behavioral correlate of the gamma oscillations in the 20–50 Hz range observed in cortical structures (Schroeder and Lakatos, 2009).

Regardless of whether the “windowed sampling” hypothesis is ultimately shown to be correct or incorrect, it speaks to the importance of performing behavioral experiments that disambiguate the roles of spatial vs. timing cues during vibrissotactile exploration, analogous to questions currently being asked in the psychophysical literature for the human hand.

## Funding

This work was supported by National Science Foundation awards IOS-0818414, IOS-0846088, EFRI-0938007 and National Institute of Health award R01-NS093585 to MH. RT was supported in part by a NIH Ruth L. Kirschstein National Research Service Award.

### Conflict of interest statement

The authors declare that the research was conducted in the absence of any commercial or financial relationships that could be construed as a potential conflict of interest.
